# Reproductive history and breast cancer risk

**DOI:** 10.1007/s12282-012-0384-8

**Published:** 2012-06-19

**Authors:** Shunzo Kobayashi, Hiroshi Sugiura, Yoshiaki Ando, Norio Shiraki, Takeshi Yanagi, Hiroko Yamashita, Tatsuya Toyama

**Affiliations:** 1 Department of Breast and Endocrine Surgery, Nagoya City West Medical Center, 1-1-1, Hirate-cho, Kita-ku, Nagoya, 462-8508 Japan; 2 Department of Diagnostic Radiology, Nagoya City West Medical Center, Nagoya, Japan; 3 Department of Radiotherapy, Nagoya City West Medical Center, Nagoya, Japan; 4 Department of Breast and Endocrine Surgery, Hokkaido University Hospital, North 14, West 5, Kita-ku, Sapporo, 060-8648 Japan; 5 Department of Oncology, Immunology and Surgery, Nagoya City University Graduate School of Medical Sciences, 1-Kawasumi, Mizuho-ku, Nagoya, 467-8601 Japan

**Keywords:** Breast cancer risk, Reproductive history, First full-term birth, Post lactation involution, Breast feeding

## Abstract

The fact that reproductive factors have significant influence on the risk of breast cancer is well known. Early age of first full-term birth is highly protective against late-onset breast cancers, but each pregnancy, including the first one, increases the risk of early-onset breast cancer. Estradiol and progesterone induce receptor activator of NF-kappa B ligand (RANKL) in estrogen receptor (ER)- and progesterone receptor (PgR)-positive luminal cells. RANKL then acts in a paracrine fashion on the membranous RANK of ER/PgR-negative epithelial stem cells of the breast. This reaction cascade is triggered by chorionic gonadotropin during the first trimester of pregnancy and results in the morphological and functional development of breast tissue. On the other hand, the administration of non-steroidal anti-inflammatory drugs in the early steps of weaning protects against tumor growth through reduction of the acute inflammatory reaction of post lactation remodeling of breast tissue. This is experimental evidence that may explain the short-term tumor-promoting effect of pregnancy. The protective effect of prolonged breast feeding may also be explained, at least in a part, by a reduced inflammatory reaction due to gradual weaning. Delay of first birth together with low parity and short duration of breast feeding are increasing social trends in developed countries. Therefore, breast cancer risk as a result of reproductive factors will not decrease in these countries in the foreseeable future. In this review, the significance of reproductive history with regard to the risk of breast cancers will be discussed, focusing on the age of first full-term birth and post lactation involution of the breast.

## Introduction

The classification of breast cancers according to the molecular profile of gene expression patterns has improved the fundamental understanding and the rational strategy of research and treatment of patients with breast cancer [1]. Breast cancers have been classified into 5 subtypes: luminal-A, luminal-B, Her2-enriched, basal-like, and claudin-low [2]. The alternative but practical classification using immunohistochemical staining of estrogen receptor (ER), progesterone receptor (PgR), and Her2 protein has been used worldwide [3]. Hormone dependency, per se, is a well-known important characteristic of breast cancers [4]. Therefore, the actual treatment of patients with breast cancer has often been based on the cancer subtype classification [5].

On the other hand, individual patient differences, especially regarding their hormonal environment due to age, menopausal status, pregnancy, and/or lactation, have also been considered mainly from the viewpoint of diagnosis and treatment [6]. However, the fundamental and clinical importance of postpartum involution of breast tissues as a tumor-promoting risk factor has recently received a great deal of attention [7, 8]. Expression of inflammation-related genes, such as COX2 [9], Stat3 [10], and tenascin C [11, 12], has been investigated, and the suppression of the resulting inflammatory reactions with non-steroidal anti-inflammatory drugs (NSAIDs) was reported to protect against tumor invasion and metastasis [13]. Thus, postpartum involution, together with the molecular subtypes may have a significant impact on the prognosis of patients, especially those who are premenopausal.

It is well known that pregnancy and breast feeding have dual effects on breast cancer development [14, 15]. Although early age of first full-term birth is highly protective against late occurrence of hormone-dependent breast cancers [16–19], each successive pregnancy of multiparous women has a progressive effect on breast cancers irrespective of hormone dependency [20]. This review will discuss the significance of pregnancy and lactation as risk factors for breast cancer.

## Age of first full-term birth

In a worldwide case–control study, MacMahon [14] was the first to report the protective effect of early age of first full-term birth against breast cancer. Compared to nulliparous women, mothers with their first full-term birth before 20 years of age had a 50 % reduced risk of breast cancer. On the other hand, those who had their first baby after age 35 had a 22 % increased risk. These relative risks were comparable across countries. The protective effect of early age of first full-term birth in parous women was similarly observed in other series from the USA [21] and Japan [22]. Except for one study from Japan [23], many reports observed a protective effect of early age of first full-term birth on hormone receptor-positive cancers [16, 18, 19, 24]. A meta-analysis of 9 cohort or case–control studies also revealed a reduced risk among patients with hormone receptor-positive cancers [17]. This protective effect was also observed in lobular carcinomas that are often positive for hormone receptors [25]. In this context, the relative protective effect of young age of first full-term birth has been observed mainly for postmenopausal women [17, 23, 24, 26].

Parity-related functional and morphological changes of breast tissue have been extensively studied by Russo and co-workers. The gene expression signature and differentiation-related chromatin remodeling in the breast tissues of parous women were distinct from those of nulliparous women [27]. Chorionic gonadotropin is secreted mostly during the first trimester of pregnancy and stimulates the ovarian granulosa-lutein cells to form the corpus luteum of pregnancy. The high levels of progesterone from the corpus luteum are critical to regulate the initial stages of the pregnancy, including breast development to prepare for a fully functional organ. Human chorionic gonadotropin (hCG) administration induced differentiation-related gene expression changes in breast epithelial cells in culture [28], and the profiles were similar in pregnancy and after hCG administration [29, 30]. On the other hand, a prolonged suppressive effect on carcinogen-induced mammary tumor formation was observed with the administration of hCG in rats [31]. A similar protective effect of hCG was observed in humans in a case–control study from Sweden [32]. Women over 50 years of age with higher blood levels of hCG during their first full-term pregnancy showed a 33 % reduced risk of breast cancer. A possible decrease in the number of mammary epithelial stem cells after pregnancy was reported in transplantation experiments [33]. Thus, the high progesterone conditions induced by the hCG seem to be essential to pregnancy-associated breast development, presumably as a result of mammary epithelial stem cell expansion.

**Fig. 1:**
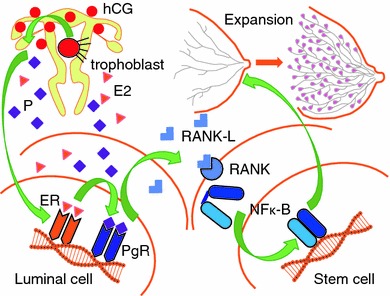
Hormonal induction of stem cell expansion for lactation triggered by hCG from the trophoblast. *hCG* human chorionic gonadotropin, *E2* estradiol, *P* progesterone, *ER* estrogen receptor, *PgR* progesterone receptor, *NFk-B* nuclear factor kappa B, *RANK* receptor activator of NFk-B, *RANKL* RANK ligand

Progesterone acts after binding to the PgR, which is in turn induced by estrogen action via ER binding. There had been, however, great controversy about the presence or absence of ER and PgR in mammary epithelial stem cells. Chimeric ER-positive and ER-negative cells were demonstrated using a biostaining technique in ER−/− mouse mammary tissue by the transplantation of cells from wild-type mice [34]. Unexpectedly, the colony forming ability that was assumed to be one of the stem cell characteristics was distinctly observed not in the ER/PgR-positive luminal cell population, but in the negative basal cell population [35]. In 2010, the detailed mechanisms of hormone action on the development of mammary tissues during pregnancy were clarified by 2 different groups [36, 37] (Fig. 1). Elevated progesterone, which is induced by the increased hCG from the trophoblast after the first trimester of pregnancy, acts on the ER/PgR-positive luminal cells and induces receptor activator of NF-kappa B ligand (RANKL). RANKL acts in a paracrine fashion on the ER/PgR-negative mammary stem cells that express RANK on their cell membrane and promotes expansion through NF-kappa B activation. This relationship of proliferation between ER/PgR-positive and -negative cells was also observed in hormone-dependent mammary carcinogenesis [38, 39] and metastasis [40]. In the case of breast cancer, RANKL may act as an autocrine fashion, or the RANK–RANKL pathway may act without the intervention of progesterone. However, there is fundamental consensus about the significance of this pathway at least in the case of breast expansion associated with pregnancy.

## Post lactation involution and risk of breast cancer

Early in 1980, Woods and co-workers reported an important observation about parity and breast cancer. Among 371 consecutive patients with breast cancer, the mean age of diagnosis of parous women (57.9) was significantly younger than that of nulliparous women (63.1). Additionally, the mean age of diagnosis fell with increasing parity and almost 40 % of women with 3 or more children were diagnosed at less than 50 years of age [15]. This observation suggested the protective effect of parity on late-onset (postmenopausal) breast cancers together with a promoting effect on cancers occurring at younger ages (premenopausal). A transient postpartum increase in breast cancer was also reported in women younger than 50 years old [41], and premenopausal women [42]. A meta-analysis of 8 reports from northern European countries revealed that a higher age of first childbirth correlated with a higher risk of breast cancer between the ages of 35 and 54 [43]. The risk of breast cancer was increased until 15 years post delivery and decreased thereafter. The transient risk increase was more prominent in women with their first full-term birth at age 35 or older [44].

The worse prognosis of breast cancers diagnosed during pregnancy or lactation had been explained as being due to the difficulty of diagnosis and treatment [6, 20, 45, 46]. However, prognosis of those occurring shortly after delivery seemed to be even worse. Survival rates improved with increasing interval between their last birth and diagnosis [47]. The worse prognosis was more significant in patients younger than 35 years old [48]. The dual effect of pregnancy was also confirmed by a cohort study [49]. Very interestingly, women with higher blood hCG levels during the first trimester of their first full-term pregnancy had a higher risk of breast cancer occurring at a young age (age less than 40; low hCG vs. high hCG, 1.00 vs. 1.78) or shortly after delivery (less than 10 years; low hCG vs. high hCG, 1.00 vs. 4.33) [32].

The terminal duct lobular units (TDLUs) of breast tissue decrease in size and number during age-related involution. Histologically demonstrated spontaneous involution was correlated with a reduced risk of late-onset breast cancers [50]. However, since the breast tissues must provide for a possible next pregnancy after weaning, the post lactation involution of the breast is distinct from that of aging. The process begins with a cell death phase and progresses to the tissue remodeling phase [51]. Gene expression profiling revealed the induction of the Stat3-mediated cell death-related pathway at the initial step, followed by an acute phase inflammatory reaction [7, 52]. The involuting breast tissues accumulated fibrillar collagen and stimulated migration and metastasis of inoculated breast cancer cells [8]. The prognostic significance of tumor-associated macrophages has been reported in patients with breast cancer [53]. Accumulated collagen acts as an extracellular matrix mediator to recruit a tumor-promoting subtype of macrophage in the involuting breast tissues [54]. The macrophage and mast cell infiltration was observed at the late phase of involution with a chronic inflammatory reaction [10] which is controlled by TIMP3 via TNF expression [55].

Tenascin C is an extracellular matrix glycoprotein often upregulated in breast cancers [56]. The downregulation of tenascin C acts as a suppressor of the epithelial to mesenchymal transition, which is important in tumor invasion and metastasis [57]. Tenascin C suppresses Stat5 signaling and stimulates the WNT and NOTCH pathways. It is abundantly expressed at the metastatic front facilitating migration and metastasis of the tumor-initiating cells [12]. Significant accumulation of tenascin C together with collagen fiber in the involuting breast has been observed [11]. The administration of NSAIDs suppresses the progression of breast cancer cells through downregulation of tenascin C expression. These specific characteristics of the post lactation involution of breast tissue that make it a risk factor for cancer were reviewed by Lyons and co-workers who suggested the importance of an inflammatory reaction [9]. COX2 expression was stimulated not only breast tissue itself but also in inoculated breast cancer cells [13]. NSAIDs administration suppressed the COX2 expression and decreased the deposition of fibrillar collagen, resulting in a decline of tumor progression and metastasis.

Kreuzaler and co-workers reported a new concept about the Stat3-mediated cell death-related pathway at the initial step of the post lactation involution [58]. Stat3 induced lysosomal protease independently from the caspase-dependent apoptotic pathway and increased lysosomal membrane permeability. The increased lysosomal proteases digest cell membranes and induce cell death without nuclear fragmentation that is typical in apoptosis. They proposed, therefore, a necrosis type of new programmed cell death pathway in this physiological but special tissue remodeling procedure of post lactation involution.

## Breast feeding

Additional important factors relating to pregnancy are breast feeding and number of pregnancies. A longer duration of breast feeding is correlated with a lower risk of breast cancer [16, 59]. Although the risk-reducing effect has been observed only for premenopausal women in reports of small series it was also observed for pre- and postmenopausal women in a large series [16] and in a meta-analysis of 47 reports from all over the world [59]. Periodic influence of estrogen/progesterone on breast tissue can be postponed by prolonged breast feeding or increased number of pregnancies. If this hormonal environment of the post delivery period influences the risk of breast cancer, it should be restricted to ER/PgR-positive cancers. However, the protective effect was observed in both ER/PgR-positive and -negative cancers [16]. Instead, longer duration of breast feeding reduced the risk of triple-negative cancers but not of luminal cancers defined by immunohistochemical staining in premenopausal patients [60]. Since data typically define the length of breast feeding as total lifetime duration, it was correlated with the number of pregnancies, but they were not necessarily identical [59]. The significance of the number of pregnancies as a risk-reducing factor has been controversial [59, 61]. However, the data in multiparous women limited to those with more than 3 children [26] or more than 5 children [62] showed significant risk-reducing effects. Interestingly enough, the duration of breast feeding per child was also inversely related to risk of breast cancer [63, 64]. Although it has not yet been adequately studied why the prolonged breast feeding is protective against breast cancer, these findings suggest a close relationship between slow-paced weaning and mitigation of inflammatory reaction during the involution. O’Brien and co-workers observed the coexistence of 2 kinds of lobules in the breast tissue from gradually weaning mothers, suggesting a stepwise turnover of TDLUs. One type was actively lactating and the other was involuting, as indicated by infiltration of CD45-positive acute reactive immune cells and CD68-positive macrophages [54].

## Perspectives

**Fig. 2:**
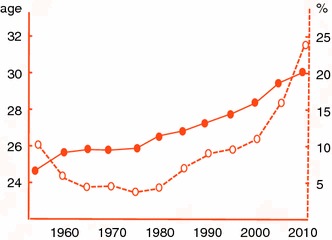
Annual trends of age of first birth (*solid line* with *solid circle*) and proportion with delivery after age 35 (*dotted line* with *open circle*) in Japan

Breast cancer incidence had been significantly lower in Japan compared to America and European countries and the rates of ER/PgR-positive breast cancers from Japanese patients were also lower, especially in postmenopausal women [65–67]. The average age of first birth has increased continuously during the past 5 decades from 24.7 to 29.7. The age-specific incidence of birth has gradually shifted to a higher value and about 24 % of women who gave birth were older than 35 years of age in 2010 (Vital Statistics Japan: Ministry of Health, Labour and Welfare) (Fig. 2). These trends must have influenced the risk of postmenopausal hormone-dependent tumors later in life as well as the risk of more aggressive tumors found shortly after childbirth. Actually, the nationwide occurrence of these two events in Japan has increased significantly during the past 3 decades in number and rates from 11,000 and 20/100,000 to 54,000 and 80/100,000, respectively [68]. The rates of cancers positive for both receptors have also increased from 50 % to more than 80 % [69].

Russo and co-workers suggested a preventive effect of periodic administration of hCG early in life on late-onset breast cancers [70]. It seems, however, to be extremely difficult to demonstrate efficacy and safety concomitantly while resolving the ethical issues. On the other hand, the administration of NSAIDs from the beginning of weaning to reduce the inflammatory reaction of involution may be applicable to prevent early-onset breast cancers. NSAIDs have been used in a wide variety of conditions and the accompanying side effects and their treatment are well known. Compared to evaluating the use of hCG, evaluation of the use of NSAIDs will require shorter follow-up periods. However, the actual significance of these agents with regard to breast cancer prevention in humans is still theoretical, or at best experimental. Methods other than administration of selective estrogen receptor modulators (SERMs) were not recommended at the present stage of knowledge in a consensus statement about preventive therapy for breast cancer by experts on breast cancer prevention in 2011 [71].

**Fig. 3:**
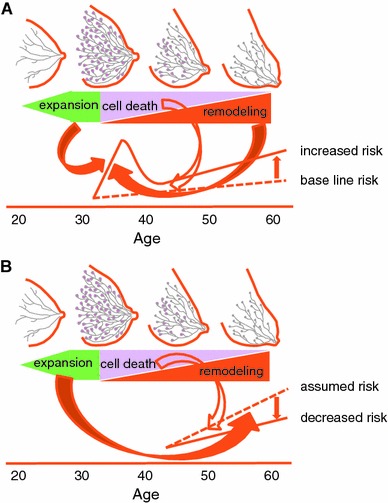
Dual effect of birth and lactation on breast cancer risk. **a** Effects on the early-onset unspecified cancer, **b** effects on the late-onset hormone-dependent cancer. *Solid arrows* promotive, *open arrows* suppressive effects on the risks

The sequence of events appearing in the lactating breast from pregnancy until weaning affects the risk of breast cancer in two ways (Fig. 3a, b). First, the cell death procedure acts in a suppressive manner. Second, the expansion and tissue remodeling procedure acts in a promotive manner. Regardless of hormone dependency, the inevitable risk of breast cancer as a female mammal become obvious around 40 years of age in humans. Among them the risk of hormone-dependent cancer is influenced largely by a variety of reproductive factors and becomes a major risk at almost 50 years of age and thereafter. On the other hand, since the first full-term birth has the most prominent dual effects on the breast cancer risk, the age when it occurs is particularly important. Early age of menarche increases the risk of ER/PgR-positive cancers [17, 72], and a long interval between menarche and first birth increases the risk by 50 % in ER/PgR-positive cancers [73]. These facts suggest an inevitable accumulation of stem-progenitor cells with unrepaired DNA damage since the initiation of pubertal development. Although these cells would have increased concomitantly during the mammary proliferation and differentiation in the first full-term pregnancy, they will be largely eliminated during the programmed cell death phase of involution after weaning. This can lead to risk reduction of late-onset hormone-dependent tumors. On the other hand, the tumor-promoting environment of the remodeling phase of involution increases the risk of unspecified tumors. The baseline risk of breast cancer is very low during younger ages. The protective effect, therefore, will be emphasized and the progressive effect will be attenuated by early age of first full-term birth. On the other hand, late age of first full-term birth inevitably increases the baseline risk of unspecified breast cancers. Although multiple consecutive pregnancies at a young age protect against late-onset cancers, this pattern in the elderly increases premenopausal cancers [74]. It is, therefore, suggested that the promotive effect is offset by the protective effect in those women with births at older ages.

Factors affecting the risk of breast cancer are diverse and are not limited to reproductive history [75]. However, because of the aging of the entire population and low fertility rates, women will be indispensable to the workforce in developed countries including Japan. Such a working environment inevitably favors the delay of the first birth together with low parity and a short duration of breast feeding. Therefore, breast cancer risk due to the increasing effects of reproductive factors will not decrease in those countries in the foreseeable future. It is thus particularly important to take into account the patient’s specific reproductive history in the diagnosis and treatment of breast cancers.
